# Gonadal steroids regulate the expression of aggrecanases in human endometrial stromal cells *in vitro*

**DOI:** 10.1111/jcmm.12110

**Published:** 2013-08-15

**Authors:** Jiadi Wen, Hua Zhu, Peter CK Leung

**Affiliations:** a Department of Obstetrics and Gynecology, University of British ColumbiaVancouver, BC, Canada

**Keywords:** aggrecanases, ADAMTS, endometrium, steroids, progestins, androgens, oestrogens

## Abstract

The human endometrium undergoes cyclic change during each menstrual cycle in response to gonadal steroids. Proteolysis of endometrial extracellular matrix (ECM) is necessary to prepare this dynamic tissue for pregnancy. Proteolytic enzymes such as matrix metalloproteinase (MMP) and closely related a disintegrin and metalloproteinase with thrombospondin motifs (ADAMTS) have been assigned key roles in the highly regulated cyclic remodelling of the endometrial ECM. We have previously shown that ADAMTS-1 undergoes spatiotemporal changes in human endometrial stromal cells under the regulation of gonadal steroids. This suggests that other ADAMTS subtypes, known as aggrecanases, may contribute to the ECM remodelling events that occur in female physiological cycles and in preparation for pregnancy. To determine whether progesterone (P4), 17β-estradiol (E2), or dihydrotestosterone (DHT), alone or in combination, are capable of regulating ADAMTS-4, -5, -8 or -9 expression in human endometrial stromal cells *in vitro*. Real-time quantitative PCR and Western blot analysis were used to measure ADAMTSs mRNA and protein levels in primary cultures of human endometrial stromal cells (*n* = 12). P4, DHT but not E2 have regulatory effects on ADAMTS-8, -9 and -5 expression. Combined treatment with gonadal steroids did not show any synergistic or antagonistic effects. However, the synthetic steroid antagonists RU486 and hydroxyflutamide specifically inhibited the P4- or DHT-mediated regulatory effects on ADAMTS expression. These studies provide evidence that the regulation of aggrecanases by gonadal steroids in human endometrial stromal cells may play an important role during decidualization.

## Introduction

Gonadal steroids govern the developmental fate of the human endometrium across the reproductive lifespan and into menopause [1, 2]. The series of progressive cellular events that occur in the endometrium in preparation for pregnancy are orchestrated by fluctuations in the physiological levels of oestrogens (estradiol; E2), progestagens (progesterone; P4), and, to a lesser extent, androgens [3, 4]. Any disruption in the bioavailability of these gonadal steroids during the menstrual cycle often results in poor reproductive outcome and menstrual disorders.

Remodelling of the endometrial ECM is a key event underlying the morphological and functional changes of endometrium and in the regulation of trophoblast invasion [5–7]. The biological action of gonadal steroids, in terms of regulating ECM remodelling-related genes associated with endometrial physiology and pathologies, remains to be elucidated. The proteolytic enzymes that are important to steroid-mediated ECM remodelling include the urokinase plasminogen activator (uPA) and the matrix metalloproteinases (MMPs) [8, 9]. However, it is important to define the full repertoire of proteinases expressed by the endometrium, their regulation, and ultimately, their individual contribution(s) to the development of a uterine environment capable of supporting pregnancy.

The ADAMTS (A Disintegrin and Metalloproteinase with ThromboSpondin repeats) are a novel family of secreted zinc-dependent metalloproteinases composed of at least 19 genetically distinct members in humans [10]. The majority of ADAMTS subtypes have been characterized at the structural level, and their expression is associated with prenatal and post-natal growth, the onset and progression of cancer, arthritis, Alzheimer's disease and a number of inflammatory and thrombotic conditions [10–12]. Some ADAMTS subtypes, such as ADAMTS-1, -4, -5, -8, -9 and -15 have been subclassified as aggrecanases because of their ability to cleave large chondroitin sulphates that provide the scaffold for ECM organization in a wide array of tissues [11]. To date, the expression, regulation and function of aggrecanases in the steroid-mediated ECM remodelling events that occur in the endometrium remain poorly characterized. We have determined that two members of the aggrecanases subfamily, *ADAMTS-1* and *-5*, are spatiotemporally expressed in the human endometrium during pregnancy [13, 14]. In addition, gonadal steroids and antisteroidal compounds exhibit complex regulatory effects on *ADAMTS-1* expression in endometrial stromal cells [15]. These results strongly suggest that gonadal steroids may regulate other ADAMTS subtypes in the human endometrium; therefore, we examined the ability of gonadal steroids to regulate the mRNA and protein levels of these ADAMTS subtypes in primary cultures of human endometrial stromal cells. In addition, we also determined whether antisteroidal compounds are capable of inhibiting the observed gonadal steroids regulatory effects on ADAMTSs expression.

## Materials and methods

### Tissues

Endometrial tissue samples were obtained from women (*n* = 12) 35–45 years old undergoing a hysterectomy for reasons other than endometrial cancer or hyperplasia in accordance with a protocol for use of human tissues approved by the Committee of Ethical Review of Research Involving Human Subjects, University of British Columbia. All of these women had normal menstrual cycles and did not receive hormonal treatments for ≥3 months prior to the time of surgery. Menstrual cycle stage was determined by the last menses and was confirmed by subsequent histological evaluation [1]. Only endometrial tissues obtained at the stage of the late secretory phase were used for stromal cell isolation.

### Cell isolation and culture

Enriched stromal cell cultures were isolated from endometrial tissues according to a previously described protocol [16]. Briefly, endometrial tissue samples were minced and subjected to 0.1% collagenase (type IV)/hyaluronidase (type I-S, Sigma-Aldrich, St Lois, MO, USA) digestion in a shaking water bath at 37°C for 60 min. The cell digest was then passed through a nylon sieve (38 μm), after which, the eluate containing the stromal cells was centrifuged at 800 × g for 10 min. at room temperature. The resultant cell pellet was washed once and resuspended in phenol red-free DMEM containing 25 mM glucose, L-glutamine, antibiotics (100 U/ml penicillin and 100 μg/ml streptomycin) and supplemented with 10% charcoal-stripped FBS. All the endometrial stromal cell cultures included in these studies were determined by immunocytochemical analysis, which was performed with a variety of markers, to have a purity of ≥99% [16, 17].

### Experimental culture conditions

Endometrial stromal cells (passage 4–6) were plated in 60 mm^2^ tissue culture dishes (Becton Dickinson and Co, Franklin Lakes, NJ, USA) at a density of 5 × 10^6^ cells/dish and were grown to 80% confluence. Cells were then washed with PBS and were cultured in phenol red-free DMEM supplemented with 10% charcoal-stripped FBS containing either increasing concentrations of P4 (1–5 μM), E2 (1–100 nM), or DHT (1–500 nM) for 24 hrs or a fixed concentration of P4 (1 μM), E2 (30 nM) or DHT (100 nM) for 0–72 hrs.

Combinatorial effects of gonadal steroids on ADAMTSs mRNA and protein levels were investigated by culturing stromal cells in the presence of P4 (1 μM) alone or in combination with increasing concentrations of E2 (0.1–100 nM) for 72 hrs, or with either P4 (1 μM) or DHT (100 nM) alone or in combination for 72 hrs.

To determine whether the observed regulatory effects of P4 and DHT on stromal ADAMTSs mRNA levels could be inhibited by antisteroidal compounds, endometrial stromal cells were cultured in the presence of increasing concentrations of RU486 (25 nM–10 μM) or hydroxyflutamide (0.1 nM–1 μM) alone or in combination with P4 (1 μM) or DHT (100 nM) for 72 hrs.

Endometrial stromal cells cultured with vehicle (0.1% ethanol) served as controls for these experiments. The concentrations of gonadal steroids and antisteroidal compounds examined in this study are based upon previous reports [16–18].

### RNA preparation and synthesis of first-strand cDNA

Total RNA was extracted from endometrial stromal cell cultures performed with a RNeasy Mini Kit (Qiagen, Mississauga, ON, Canada). The purity and concentration of total RNA present in each of these extracts were quantified by absorbance (260/280 nm) performed with a Du-64 UV-spectrophotometer (Beckman Coulter, Mississauga, ON, Canada).

Aliquots (∼1 μg) of total RNA extracts prepared from the endometrial stromal cell cultures were subsequently reverse transcribed into cDNA performed with a First-Strand cDNA Synthesis Kit (Amersham Pharmacia Biotech, Oakville, ON, Canada).

### Real-time quantitative (q)RT-PCR

The first-strand cDNA generated from endometrial stromal cell cultures served as a template for qRT-PCR performed with the ABI PRISM 7300 Sequence Detection System (Perkin-Elmer Applied Biosystems, CA, USA) equipped with a 96-well optical reaction plate for primers specific for *ADAMTS-4, -5, -8, -9, -15*, *Versican* or *GAPDH* (a housekeeping gene). The specific nucleotide sequences for these primers are as follows: *ADAMTS-4* Forward 5′-CACAT CCTAC GCCGG AAGAG-3′, Reverse 5′-GAGCC TTGAC GTTGC ACATG-3′; *ADAMTS-5* Forward 5′-AATAA CCCTG CTCCC AGAAA CA-3′, Reverse 5′-GCGGT AGATG GCCCT CTTC-3′; *ADAMTS-8* Forward 5′-CCAGC ATCAA GAATT CCATC AA -3′, Reverse 5′- CCCAT TTTTC ATCTT CTACG ATCA-3′; *ADAMTS-9* Forward 5′-GGAAC AAAAC AAACC CCACA TC-3′, Reverse 5′-CCTTC CTGTT GAGGG CTCTC T-3′; ADAMTS-15 Forward 5′-GGCCT GCGTG GAGAG ACA-3′, Reverse 5′-CCCAT TTGGC CCAGC AA-3′; *Versican* Forward 5′-TGAGC CTACC TTGTC ATTTT TCAAC-3′, Reverse 5′-CATTT GATGC GGAGA AATTC AC-3′; and *GAPDH* Forward 5′-TGGAA ATCCC ATCAC CATCT T -3′, Reverse 5′-CGCCC CACTT GATTT TGG-3′.

Real-time qPCR was performed with 12.5 μl SYBR® Green PCR Master Mix (Perkin-Elmer Applied Biosystems), 7.5 μl of primer mixture (300 nM), and 5 μl of cDNA template (diluted 1: 7 v/v) under the following optimized conditions: 52°C for 2 min. followed by 95°C for 10 min. and 40 cycles of 95°C for 15 sec. and 60°C for 1 min. All PCR reactions were performed in duplicate, with the mean used to determine mRNA levels. A negative control containing water instead of sample cDNA was included in each plate. Each set of primers generated a single PCR product of the appropriate size, visualized by agarose gel electrophoresis and by melt curve analysis following qRT-PCR. PCR products were sequenced and confirmed by BLAST (http://www.ncbi.nlm.nih.gov). The amplification efficiency was determined by plotting the log cDNA dilution against ΔC_T_ (ΔC_T_ = C_T.Target_ − C_T.GAPDH_), the slope of which was close to zero indicating maximal and similar efficiency of the target and reference genes (data not shown). Relative ADAMTS-4, -5, -8, and -9 mRNA levels were determined using the formula 2^−ΔΔC^_T_, where ΔΔC_T_ = (C_T.Target_ − C_T.GAPDH_)_X_ − (C_T.Target_ − C_T.GAPDH_)_0_. In this formula, X represents any time-point or experimental treatment with control cultures being assigned a value of zero [19]. Data were analysed using SDS 2.0 software (Applied Biosystems). This experimental approach was further validated by the observation that differences between the C_T_ for the target gene and GAPDH remained relatively constant for each amount of DNA examined.

### Western blot analysis

Endometrial stromal cell cultures were washed three times in PBS and were incubated in 100 μl of cell extraction buffer (Biosource International, Camarillo, CA, USA) supplemented with 1.0 mM PMSF and a protease-inhibitor cocktail for 30 min. on a rocking platform. Cell lysates were centrifuged at 10,000 × *g* for 10 min. at 4°C, and the supernatants were used for western blotting. Cell lysate protein concentrations were determined with a BCA kit (Pierce Chemicals, Rockford, IL, USA). Western blots containing cell-lysate aliquots (∼30 μg) were prepared and immunoblotted with a polyclonal antibody directed against human ADAMTS-4, -5, -8 and -9 (Biodesign International, Saco, ME, USA). To standardize the amounts of protein loaded into each lane, the blots were reprobed with a polyclonal antibody directed against human β-actin (Sigma-Aldrich). The Amersham ECL system was used to detect the amount of each antibody bound to antigen, and the resultant photographic films were analysed by UV densitometry (GE Healthcare Life Sciences, Pittsburgh, PA, USA). The absorbance values obtained for ADAMTSs were then normalized relative to the corresponding β-actin absorbance value.

### Statistical analysis

Absorbance values obtained by qRT-PCR were subjected to statistical analysis performed with GraphPad Prism 4 computer software (GraphPad, San Diego, CA, USA). Statistical differences between the absorbance values were assessed by anova. Differences were considered significant for *P* < 0.05. Significant differences between the means were determined with Dunnett's test.

## Results

### Expression levels of aggrecanases and versican in endometrial stroma and first trimester decidual cells

**Fig. 1:**
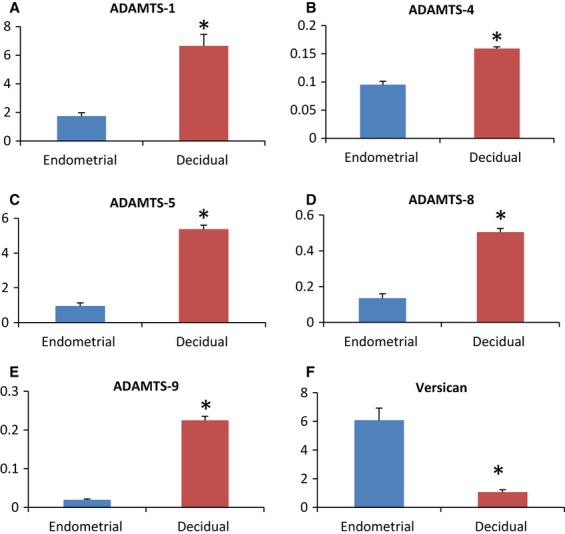
Comparison of aggrecanases and versican mRNA levels in primary culture of human endometrial stromal cells and first trimester decidual stromal cells. Real-time qRT-PCR analysis of ADAMTS-1, -4, -5, -8, -9 and their common substrate Versican (**A**–**F**), mRNA levels in primary cultures of human endometrial stromal cells compared with first trimester endometrial decidual cells. Values for ADAMTS mRNA levels present in each sample were normalized to GAPDH mRNA levels. The results are derived from at least four sets of samples and are represented (mean ± SEM; *n* ≥ 4) in the bar graphs (*P < 0.05, first trimester decidual stromal cells *versus* endometrial stromal cells).

*ADAMTS-1, -4, -5, -8, -9* and *Versican* mRNA are expressed in primary cultures of endometrial stromal cells and first trimester decidual stromal cells (Fig. 1), but *ADAMTS-15* mRNA was not detectable in either cell culture (data not shown). In decidual stromal cells, *ADAMTS-1* and *-8* mRNA levels are ∼fourfold higher (Fig. 1A and D), *ADAMTS-5* mRNA levels are about sixfold higher (Fig. 1C), and *ADAMTS-9* mRNA levels are about 11-fold higher than in endometrial stromal cells (Fig. 1E). In contrast, *Versican* mRNA levels are ∼sixfold lower in decidual cells (Fig. 1F).

### Distinct regulatory effects of gonadal steroids on ADAMTS-4, -5, -8 and -9 mRNA and protein levels in human endometrial stromal cells *in vitro*

**Fig. 2:**
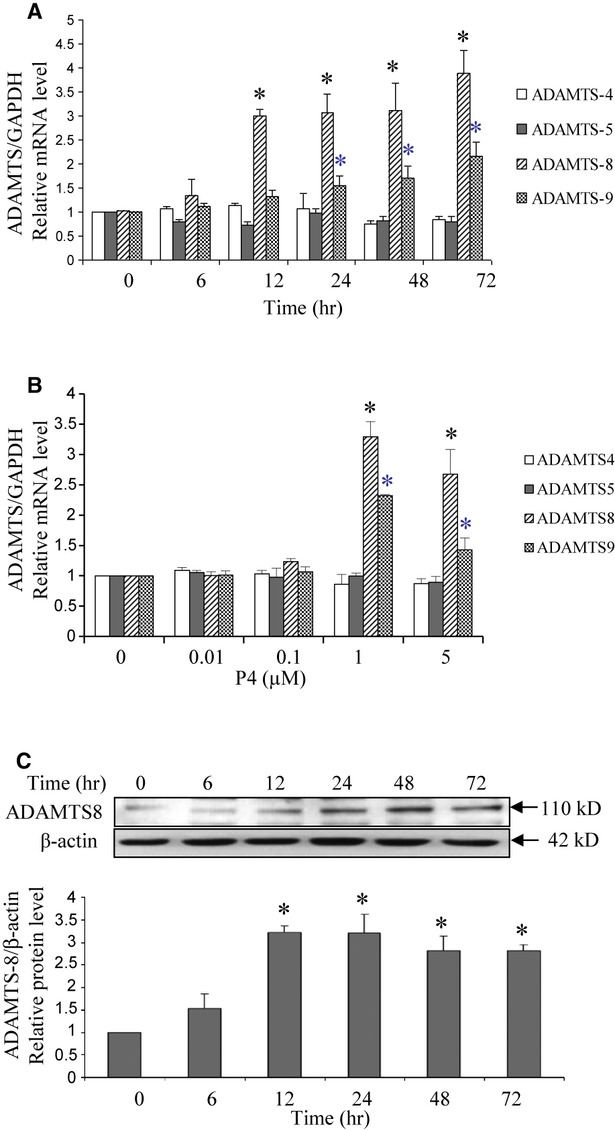
Regulatory effects of P4 on aggrecanase mRNA and protein levels in human endometrial stromal cells. (**A**, **B**) Real-time qRT-PCR analysis of ADAMTS-4, -5, -8 and-9 mRNA levels in human endometrial stromal cells. (**A**) Cells cultured in the presence of P4 (1 μM) for 0–72 hrs. (**B**) Concentration-dependent effects of P4 (0–5 μM) on ADAMTSs mRNA levels in cells cultured for 72 hrs. Values for the ADAMTSs mRNA levels present in each sample were normalized to GAPDH mRNA levels. (**C**) Representative western blots containing total protein extracted from endometrial cell cultures treated with P4 (1 μM) for 0–72 hrs. Cell lysates (30 μg) were analysed by SDS-PAGE and immunoblotted. The top half of the membrane was probed with rabbit polyclonal antibodies directed against ADAMTS-8, and the bottom half was probed for human β-actin. The Amersham ECL system was used to detect antibody bound to antigen. ADAMTS-8 protein levels were normalized to absorbance values obtained for β-actin. The mRNA and protein results were derived from at least four sets of samples, were standardized to the untreated controls, and are represented (mean ± SEM; *n* ≥ 4) in the bar graphs (*P < 0.05 *versus* untreated control).

Progesterone (P4) significantly increased *ADAMTS-8* and *-9* mRNA levels in stromal primary cell cultures in a concentration- and time-dependent manner (Fig. 2). The addition of vehicle (ethanol) alone to the culture medium had no significant effect on aggrecanase mRNA levels at any of the time-points examined (data not shown). P4 significantly increased *ADAMTS-8* and *-9* mRNA levels after 12 h and 24 h of incubation at the concentration of 1 μM (Fig. 2A), and these levels continued to increase until the termination of the experiment at 72 hrs. In addition, *ADAMTS-8* and *-9* mRNA levels were significantly increased after treatment with high concentrations (1 and 5 μM) of P4 (Fig. 2B). In contrast, P4 had no effects on the *ADAMTS-4* and *-5* mRNA levels.

Moreover, 17-β estradiol (E2) treatment had no effect on ADAMTS-4, -5, -8 and -9 mRNA levels at any of the time-points and concentrations examined in this study (data not shown).

**Fig. 3:**
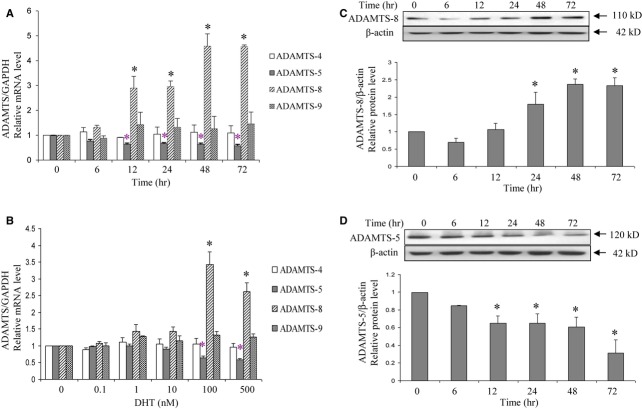
Regulatory effects of DHT on aggrecanase mRNA and protein levels in human endometrial stromal cells. (**A**, **B**) Real-time qRT-PCR analysis of ADAMTS-4, -5, -8 and-9 mRNA levels in human endometrial stromal cells. (**A**) Cells cultured in the presence of DHT (100 nM) for 0–72 hrs. (**B**) Concentration-dependent effects of DHT (0–500 nM) on ADAMTSs mRNA levels in cells cultured for 72 hrs. Values for ADAMTSs mRNA levels present in each sample were normalized to GAPDH mRNA levels. (**C**, **D**) Representative western blots containing total protein extracted from endometrial cell cultures treated with DHT (100 nM) for 0–72 hrs. Cell lysates (30 μg) were analysed by SDS-PAGE and immunoblotting. The top half of membrane was probed with rabbit polyclonal antibodies directed against (**C**) ADAMTS-8 or (**D**) ADAMTS-5, and the bottom half was probed for human β-actin. The Amersham ECL system was used to detect antibody bound to antigen. Values for ADAMTS-5 and -8 were normalized to absorbance values obtained for β-actin. The mRNA and protein results derived from at least four sets of samples were standardized to the untreated controls and are represented (mean ± SEM; *n* ≥ 4) in the bar graphs (*P < 0.05 *versus* untreated control).

The non-aromatizable androgen, DHT, significantly increased *ADAMTS-8* mRNA levels in a time- and concentration-dependent manner (Fig. 3). The increase in *ADAMTS-8* mRNA was detectable 12 hrs after treatment with 100 nM DHT, increased fourfold at 48 hrs, and was maintained for at least 72 hrs of treatment. Dihydrotestosterone treatment decreased the amount of *ADAMTS-5* mRNA by about 40% after 12 hrs of treatment (Fig. 3A). The DHT-induced regulatory effects on *ADAMTS-8* and *-5* mRNAs were observed with high concentrations (100 and 500 nM) of DHT after 72 hrs treatment, but not with concentrations less than 10 nM (Fig. 3B).

Preliminary data showed that the expression of ADAMTS-4 and -9 proteins were barely detectable, and therefore this study focused on characterizing the protein levels of ADAMTS-5 and ADAMTS-8. The ADAMTS-8 and ADAMTS-5 zymogens (110 and 120 kD respectively) were detectable in whole lysates from cultured endometrial stromal cells. ADAMTS-5 and -8 zymogens levels remained relatively constant in endometrial stromal cells treated with vehicle alone (data not shown). P4 and DHT, but not E2, significantly increased the amount of ADAMTS-8 protein by ∼threefold, which was detectable 12–24 hrs after treatment and extended to at least 72 hrs after treatment (Figs 2C and 3C). In accordance with the *ADAMTS-5* mRNA measurements, ADAMTS-5 protein levels in the endometrial stromal cells decreased significantly in a time-dependent manner after treatment with DHT (Fig. 3D).

### Combinatorial effects of gonadal steroids on stromal ADAMTS-5, -8 and -9 mRNA and protein levels

**Fig. 4:**
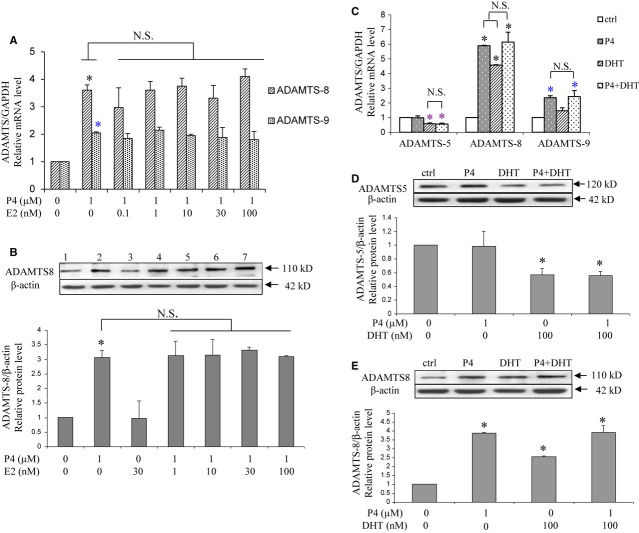
Combinatorial effects of gonadal steroids on stromal ADAMTS-5, -8, and -9 mRNA and protein levels. (**A**, **B**) Endometrial stromal cells were treated with P4 (1 μM) alone or in combination with increasing concentrations of E2 (0.1, 1, 10, 30, 100 nM) for 72 hrs. (**A**) Real-time qRT-PCR analysis of ADAMTS-8 and -9 mRNA levels. (**B**) Western blot analysis of ADAMTS-8 expression in protein extracts (30 μg) prepared from the corresponding cell cultures. While all treated samples showed significant increases (*P < 0.05) in ADAMTS-8 and -9 mRNA and ADAMTS-8 protein levels compared with untreated samples, no significant differences (N.S.) were found when E2 was present with P4 compared to P4 alone. (**C**–**E**) Endometrial stromal cells were cultured in the presence of P4 (1 μM) or DHT (100 nM) alone or in combination for 72 hrs. (**C**) Real-time qRT-PCR analysis of ADAMTS-5, -8 and -9 mRNA levels. (**D**) Western blot analysis of ADAMTS-5 expression in protein extracts (30 μg) prepared from the corresponding cell cultures. (**E**) Western blot analysis of ADAMTS-8 expression in protein extracts (30 μg) prepared from the corresponding cell cultures. No significant differences (N.S.) were found upon co-treatment with P4 and DHT compared to P4 or DHT alone. Values for ADAMTS-5, -8 and -9 mRNA levels present in each sample were normalized to GAPDH mRNA levels. Western blotting was performed with the Amersham ECL system to detect antibody bound to antigen. Cell lysates were analysed by SDS-PAGE and immunoblotting. The top half of the membrane was probed for ADAMTS-5 or -8, and the bottom half was probed for μ-actin. The absorbance values obtained for ADAMTS-5 or -8 were normalized to β-actin. The results were derived from at least four sets of samples, were standardized to the untreated controls, and are represented (mean ± SEM; *n* ≥ 4) in the bar graphs (*P < 0.05 *versus* untreated control; N.S., no significant difference).

Unless otherwise stated, all results were measured after 72 hrs of culture. E2 can abolish the P4-mediated increase in *ADAMTS-1* mRNA and protein levels in stromal cells [15], but increasing concentrations of E2 did not affect the P4-mediated increase in *ADAMTS-8* and *-9* mRNA levels in primary cultures of endometrial stromal cells (Fig. 4A). When cells were co-treated with E2 and P4, the ADAMTS-8 protein expression remained the same compared with cells treated with P4 alone (Fig. 4B).

A combination of P4 and DHT caused a significant increase in about fourfold and 2.5-fold in *ADAMTS-8* and *-9* mRNA levels, respectively, by 72 hrs. Increased amounts of *ADAMTS-8* and *-9* mRNAs were similar to the increase observed in endometrial stromal cells cultured in the presence of P4 or DHT alone (Fig. 4C). The amount of ADAMTS-8 protein present in these cell cultures increased about threefold, similar to what was observed after P4 treatment alone (Fig. 4E). Treatment with both P4 and DHT caused a significant decrease (about 40%) in ADAMTS-5 mRNA and protein levels, similar to what was observed in endometrial stromal cells cultured in the presence of DHT alone (Fig. 4C and D).

### Regulatory effects of antisteroidal compounds on stromal ADAMTS-5, -8 and -9 mRNA levels

**Fig. 5:**
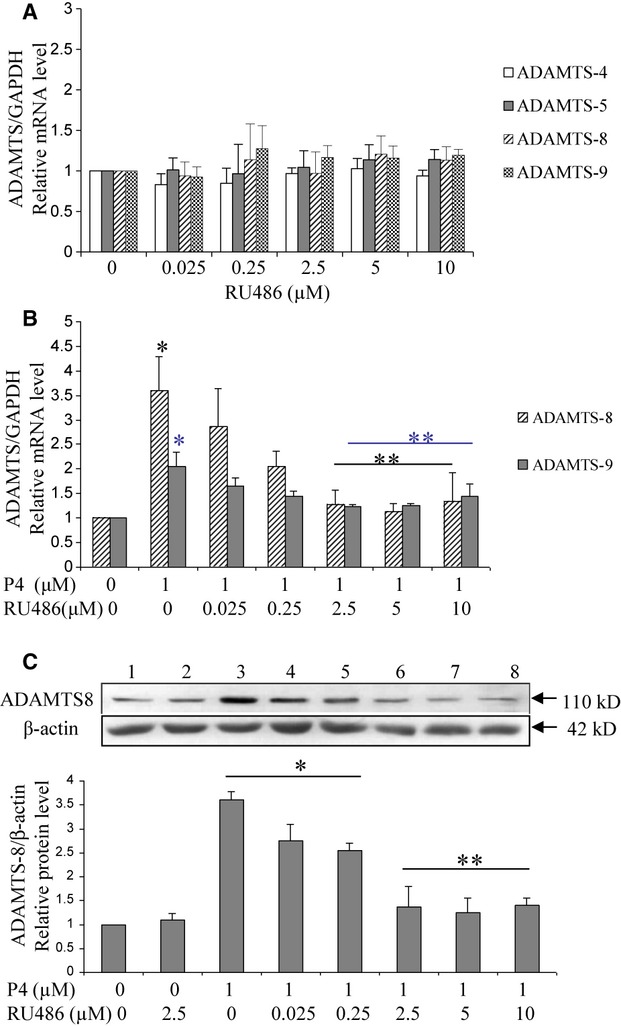
Inhibitory effects of antisteroidal compound RU486 on P4-mediated regulation of ADAMTS-8 and -9 in endometrial stromal cells. (**A**–**C**) Inhibitory effects of RU486 on P4-mediated regulation of ADAMTS-8 and -9. (**A**) Real-time qRT-PCR analysis of ADAMTS-4, -5, -8 and -9 mRNA levels in endometrial stroma cells cultured with increasing concentration of RU486 for 72 hrs. (**B**) ADAMTS-8 and -9 mRNA levels in endometrial stromal cells cultured in the presence of P4 (1 μM) alone or in combination with increasing concentrations of RU486 for 72 hrs. (**C**) Western blot analysis of ADAMTS-8 levels in protein extracts (30 μg) prepared from endometrial stromal cells cultured in the presence of P4 (1 μM) (lane 3), RU486 (2.5 μM) (lane 2), or P4 plus increasing concentrations of RU486 (lanes 4–8). Lane 1 indicates non-treatment control. Values for the ADAMTSs mRNA levels were normalized to GAPDH mRNA levels. Western blot analysis was performed with the Amersham ECL system to detect antibody bound to antigen. Cell lysates were analysed by SDS-PAGE and immunoblotting. The top half of the membrane was probed for ADAMTS-5 or -8, and the bottom half was probed for β-actin. Absorbance values obtained for ADAMTS-5 or -8 were normalized to β-actin. The results derived from at least four sets of samples were standardized to untreated controls and are represented (mean ± SEM; *n* ≥ 4) in the bar graphs (*P < 0.05 *versus* untreated control; **P < 0.05 *versus* P4 treated alone).

**Fig. 6:**
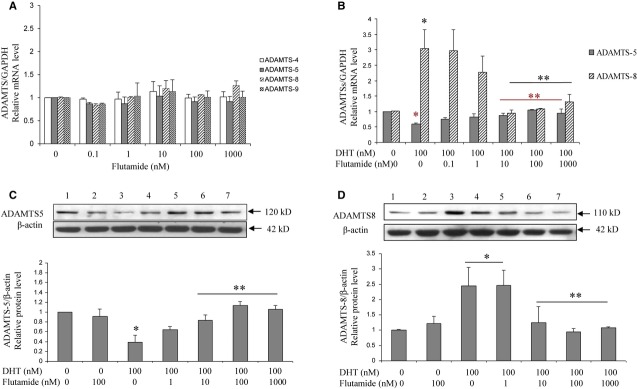
Inhibitory effects of hydroxyflutamide on DHT-mediated regulation of ADAMTS-5 and -8 in endometrial stromal cells. (**A**–**D**) Inhibitory effects of hydroxyflutamide on DHT-mediated regulation of ADAMTS-5 and -8. (**A**) Real-time qRT-PCR analysis of ADAMTS-4, -5, -8 and -9 mRNA levels in endometrial stroma cells cultured with increasing concentration of hydroxyflutamide for 72 hrs. (**B**) ADAMTS-5 and -8 mRNA levels in endometrial stromal cells cultured in the presence of DHT (100 nM) alone or in combination with increasing concentrations of hydroxyflutamide for 72 hrs. (**C**) Western blot analysis of the effects of hydroxyflutamide on DHT-mediated ADAMTS-5 expression in protein extracts (30 μg) prepared from endometrial stromal cells cultured in the presence of DHT (100 nM) (lane 3), hydroxyflutamide (100 nM) (lane 2), or DHT plus increasing concentrations of hydroxyflutamide (lanes 4–7). (**D**) ADAMTS-8 expression in protein extracts (30 μg) prepared from stromal cells cultured in the presence of DHT (100 nM) (lane 3), hydroxyflutamide (100 nM) (lane 2), or DHT plus increasing concentrations of hydroxyflutamide (lanes 4–7). Lane 1 indicates nontreatment control. Values for ADAMTSs mRNA levels were normalized to GAPDH mRNA levels. Western blot analysis was performed with the Amersham ECL system to detect antibody bound to antigen. Cell lysates were analysed by SDS-PAGE and immunoblotting. The top half of the membrane was probed for ADAMTS-5 or -8, and the bottom half was probed for β-actin. Absorbance values obtained for ADAMTS-5 or -8 were normalized to β-actin. The results were derived from at least four sets of samples, were standardized to the untreated controls, and are represented (mean ± SEM; *n* ≥ 4) in the bar graphs (*P < 0.05 *versus* untreated control; **P < 0.05 *versus* DHT treated alone).

*ADAMTS-4, -5, -8* and -*9* mRNA levels were relatively constant in endometrial stromal cells cultured in the presence of increasing concentrations of the anti-progestin, RU486, or the anti-androgen, hydroxyflutamide (Figs 5A and 6A). However, RU486 inhibited the P4-mediated increase in *ADAMTS-8* and *-9* mRNA levels in stromal cells, and hydroxyflutamide inhibited the DHT-mediated increase in *ADAMTS-8* and decrease in *ADAMTS-5* mRNA levels in stromal cells in a concentration-dependent manner. Maximal inhibition was observed at RU486 concentrations greater than 2.5 μM (Fig. 5B), and hydroxyflutamide concentrations greater than 10 nM (Fig. 6B). The P4-mediated increase in ADAMTS-8 protein levels was abolished by RU486 in accordance with *ADAMTS-8* mRNA measurements (Fig. 5C), but this effect was not inhibited by hydroxyflutamide (data not shown). Dihydrotestosterone-mediated regulation of ADAMTS-5 and -8 protein levels were inhibited by hydroxyflutamide (Fig. 5C and D), but not RU486 (data not shown).

## Discussion

Members of the ADAMTS aggrecanases subfamily (ADAMTS-1, -4, -5, -8 and -9) have been detected in a broad spectrum of human tissues including the uterus and placenta [10]. Studies have shown both redundant and non-redundant roles for ADAMTS subtypes in the reproductive system [20]. Previous studies have demonstrated that the expression of ADAMTS-1 in human endometrial stromal cells is under the control of gonadal steroids [14]. Here, we have examined the regulation of ADAMTS-4, -5, -8 and -9 mRNA and protein levels in human endometrial stromal cells by gonadal steroids.

In addition to *ADAMTS-1* [14], progesterone positively regulates the expression of *ADAMTS-8* and -*9* in human endometrial stromal cells. However, despite testing both physiological and non-physiological concentrations of progesterone, only the latter (1 or 5 μM) were able to induce changes in the expression of ADAMTS-8 and -9. Peritoneal progesterone concentrations approaching 1 μM have been described following ovulation [21], thus the local progesterone concentration in the uterus may be considerably higher than the serum levels. Nevertheless, considering the nanomolar affinity of PR for progesterone [22], and those responses were observed at concentrations exceeding the maximum serum levels of progesterone in normal women, it is not certain whether the effects of progesterone are mediated by PR. For example, glucocorticoid receptor (GR) can also bind progesterone but does so with much lower affinity than PR [23]. Thus, the responses observed with high concentrations of progesterone could reflect the regulation of ADAMTS-8 and -9 by GR. Indeed, progesterone-mediated regulation of ADAMTS-8 and -9 expression was abolished by RU486, a potent antagonist of both PR and GR.

Studies have demonstrated GR expression in the stromal compartment of the human endometrium throughout the cycle [24, 25]. Moreover, progesterone (1–10 μM) has been shown to regulate uterine natural killer cells *via* GR in the absence of PR [26]. There is also evidence to suggest that the suppressive effects of progesterone on immunocytes are mediated by GR and/or PR [27–30]. To our knowledge, the effects of glucocorticoids on aggrecanase expression have not been examined in the female reproductive system. However, studies have shown that glucocorticoids can regulate the expression of ADAMTS-4 and -5 in equine chondrocytes [31]. Moreover, glucocorticoids have been shown to regulate ADAMTS-2 expression in cell lines of monocyte lineage, but not epithelial or fibroblastic lineage, indicating tissue-specific regulation of aggrecanases by glucocorticoids [32]. In this context, our findings could suggest important roles for glucocorticoids in the regulation of aggrecanase expression during endometrial decidualization. Future studies aimed at addressing the roles of PR and GR, as well as the effects of glucocorticoids, will therefore be of great interest.

With the exception of *ADAMTS-15*, aggrecanases were expressed in the human endometrium during the menstrual cycle and first trimester of pregnancy. Progesterone and androgen, but not oestrogen, were found to exert unique as well as overlapping regulatory effects on aggrecanase expression in endometrial stromal cells. This further suggests that the aggrecanases have significant functions in the secretory endometrium, that is, during the progesterone dominant phase of the menstrual cycle. Therefore, we predict that after ovulation, when plasma progesterone levels increase, and especially during the time when decidualization occurs, the levels of ADAMTS-1, -8 and -9 will increase, helping to degrade the endometrial ECM and thereby facilitate embryo implantation.

ADAMTS-1 knockout mice are sub-fertile as a result of decreased ovulation and morphological changes in the endometrium, although some still undergo normal decidualization [33]. This may be attributed to the overlapping/compensatory functions of other ADAMTSs, particularly in, ADAMTS-5, -8 and -9. The overall amino acid sequence identity between ADAMTS-1 and ADAMTS-8 is 52%, and both are proven anti-angiogenic factors [34]. Angiogenesis is a critical event during implantation and tumour invasion, and ADAMTS-1 and -8 have been assigned this function in several kinds of tumours [35–37], but the expression and regulation of *ADAMTS-8* in reproductive tissues have not been reported. Progesterone and DHT can both increase *ADAMTS-1* and *-8* expression in human endometrial stromal cells, supporting their roles in decidualization. Androgen receptor is abundantly expressed in human endometrium, especially the endometrial stromal compartment [38, 39]. Moreover, DHT and progesterone have similar potencies on the decidua of the rodent endometrium [40]. A recent study by Kajihara *et al*. suggests that DHT enhances the process of decidualization in human endometrial stromal cells [41]. However, this study provides evidence that the biological actions of DHT on the endometrium do not simply mimic those of progesterone but are instead mediated by an independent receptor-based pathway. Although both progesterone and DHT increased ADAMTS-8 expression, only DHT induced the down-regulation of ADAMTS-5. Moreover, DHT-mediated regulation of both ADAMTS-5 and -8 was abolished by hydroxyflutamide, an androgen receptor antagonist. ADAMTS-9 has recently been reported to have an anti-angiogenic function in cancer [42]. ADAMTS-9 responds to progesterone in the primary culture of endometrial stromal cells, however, its low expression suggests a limited function in the human endometrium.

ADAMTS-5 is highly expressed in first trimester decidua [14, 15]. The response of *ADAMTS-5* to IL-1β and TGF-β1, two potent regulators of proteolytic processes at the maternal-foetal interface [13, 43, 44], suggest an important role for *ADAMTS-5* in preparing the endometrium for implantation. Although *ADAMTS-5* did not appear to be regulated by oestrogen or progesterone, androgen caused a decrease in its expression. Interestingly, aberrant androgen-AR mediated effects have been linked to infertility [3]. Therefore, excessive androgen may cause aberrant expression of certain ADAMTS subtypes, in particular *ADAMTS-5*, which could disrupt the ECM remodelling process during the menstrual cycle. However, the biological significance of androgen regulation of *ADAMTS-5* and *-8* expression in endometrial stromal cells *in vitro* remains to be elucidated as the concentrations of DHT used in this study exceed the reported physiological range of this gonadal steroid in women during a normal menstrual cycle [45, 46].

In summary, our current results demonstrate that gonadal steroids exert complex regulatory effects on the expression of aggrecanases in primary cultures of human endometrial stromal cells. This suggests that ADAMTS subtypes may play important roles in endometrial physiology. It will be interesting to observe whether mice containing null mutations for ADAMTS-1, -5 and, -8 are infertile. This observation will confirm our findings that these aggrecanases play distinct and/or compensatory roles during endometrial decidualization.
