# Expression of Concern: Gonadal Steroids Regulate the Expression of Aggrecanases in Human Endometrial Stromal Cells In Vitro

**DOI:** 10.1111/jcmm.70755

**Published:** 2025-07-28

Expression of Concern: J. Wen, H. Zhu, and P. C. K. Leung, “Gonadal Steroids Regulate the Expression of Aggrecanases in Human Endometrial Stromal Cells In Vitro,” *Journal of Cellular and Molecular Medicine* 17, no. 10 (2013): 1325–1334, https://doi.org/10.1111/jcmm.12110.

This Expression of Concern is for the above article, published online on 15 August 2013 in Wiley Online Library (wileyonlinelibrary.com), and has been issued by agreement between the journal Editor‐in‐Chief, Stefan N. Constantinescu; the Foundation for Cellular and Molecular Medicine; and John Wiley and Sons Ltd. A third party reported that three of the four lanes in the beta‐actin control bands in Figure 4D had been duplicated in Figure 4E. The third party also noted that seven of the ADAMTS8 experimental bands in Figure 5C had been duplicated in Figure 6D even though both images are intended to show different treatments. Additional investigation by the publisher confirmed these duplications and found that the ADAMTS8 band in Figure 6D was altered with high contrast compared to the same band in Figure 5C.

The authors responded to an inquiry by the publisher and stated that the ADAMTS8 image in Figure 5C was correct and that the ADAMTS8 image in Figure 6D was mistakenly replaced with the image of the other treatment during the assembly of the ADAMTS8 Western blot panels, and that adjustments to image brightness and contrast were made during figure preparation. The authors also stated that the beta‐actin images were not duplicated and were generated from separate experiments. The authors provided what they stated was the correct image for Figure 6D. However, the authors confirmed that not all of the original data for the article were available. Because the original data could not be evaluated, the journal is not able to verify the provided replacement image for the ADAMTS8 band in Figure 6D. As such, the editors are not able to validate the data included in Figures 5 and 6. The journal has decided to issue an Expression of Concern to inform and alert readers. The authors were informed of the Expression of Concern.

